# Transcranial Magnetic Stimulation Trains at 1 Hz Frequency of the Right Posterior Parietal Cortex Facilitate Recognition Memory

**DOI:** 10.3389/fnhum.2021.696793

**Published:** 2021-10-14

**Authors:** Giuseppa Renata Mangano, Massimiliano Oliveri, Daniela Smirni, Vincenza Tarantino, Patrizia Turriziani

**Affiliations:** ^1^Department of Psychology, Educational Science and Human Movement, University of Palermo, Palermo, Italy; ^2^Neuroteam Life and Science, Palermo, Italy

**Keywords:** rTMS (repetitive transcranial magnetic stimulation), recognition memory, memory retrieval, episodic memory, posterior parietal cortex

## Abstract

Neuroimaging, neuropsychological, and brain stimulation studies have led to contrasting findings regarding the potential roles of the lateral parietal lobe in episodic memory. Studies using brain stimulation methods reported in the literature do not offer unequivocal findings on the interactions with stimulation location (left vs. right hemisphere) or timing of the stimulation (encoding vs. retrieval). To address these issues, active and sham 1 Hz repetitive transcranial magnetic stimulation (rTMS) trains of 600 stimuli were applied over the right or left posterior parietal cortex (PPC) before the encoding or before the retrieval phase of a recognition memory task of unknown faces in a group of 40 healthy subjects. Active rTMS over the right but not the left PPC significantly improved non-verbal recognition memory performance without any significant modulation of speed of response when applied before the retrieval phase. In contrast, rTMS over the right or the left PPC before the encoding phase did not modulate memory performance. Our results support the hypothesis that the PPC plays a role in episodic memory retrieval that appears to be dependent on both the hemispheric lateralization and the timing of the stimulation (encoding vs. retrieval).

## Introduction

Episodic memory is subserved by a distributed cortical-hippocampal network encompassing dorsolateral prefrontal (DLPFC) and lateral parietal cortex (Teyler and Di Scenna, 1986; McClelland et al., 1995; Nadel et al., 2000; Winocur and Moscovitch, 2011; Watrous et al., 2013). Non-invasive brain stimulation techniques, such as TMS and transcranial direct current stimulation (tDCS) can provide a causal test to modulate cortico-hippocampal connectivity and to measure corresponding changes in memory performances (Kim et al., 2016).

An open question that can be usefully addressed by these techniques is the effect of the timing of the stimulation on memory recognition, i.e., in encoding or retrieval phases.

Previous TMS studies investigating the role of parietal and frontal cortex in memory encoding and retrieval often used an event related high-frequency rTMS approach, known as an interference method, that can increase the timing for the information processing or modify criteria for response decision and thus allows to deduce if the stimulated area is causally engaged in the task under investigation. rTMS studies using this approach have shown that disrupting the right DLPFC impairs retrieval of previously learned items (Rossi et al., 2001; Sandrini et al., 2003; Gagnon et al., 2010).

When exploring the role of the parietal cortex using the same method, the findings are most contrasting. A study by Rossi et al. (2006), using on-line 20 Hz rTMS to either the left or the right intraparietal sulcus during a yes/no recognition memory task for visual scenes, failed to report a significant impact of the stimulation on the memory performances of healthy controls. In contrast, an fMRI- guided rTMS approach by Manenti et al. (2010), consisting of an fMRI-based target area selection on an individual basis followed by on-line 10 Hz rTMS trains, provided for the first time evidence that the parietal cortices have a necessary role during episodic retrieval of abstract words. In their fMRI experiment, the authors found that encoding was selectively associated with an activation of the left DLPFC, whereas retrieval selectively activated the inferior parietal lobules (supramarginal and angular gyri) bilaterally, as well as the right DLPFC. Indeed, 10 Hz rTMS caused a slowing down of performance during abstract word retrieval when applied over the left inferior parietal cortex and the right DLPFC.

More recently, rTMS investigations of different left parietal regions during episodic memory retrieval documented that transient on-line 20 Hz rTMS of the angular gyrus significantly decreased item-recognition accuracy as compared with superior parietal lobe stimulation, especially for the criterion of source memory judgments (Sestieri et al., 2013).

Complementary findings on this topic are offered by studies applying tDCS or TMS off-line, i.e., before the encoding or the retrieval phase of a memory test, in order to modulate the excitability of the parietal regions as part of thecortical-hippocampal network associated to memory. An advantage of this brain stimulation approach is that it allows modulation of plasticity of the stimulated as well as of interconnected brain areas in a memory circuit (Kim et al., 2016).

Some tDCS studies suggest that increasing excitability of the parietal regions during retrieval may improve memory performance. Pisoni et al. (2015) tested the effects of anodal tDCS in bilateral temporal and parietal regions in healthy subjects performing a verbal memory recognition task requiring to make old/new judgments. Anodal tDCS to bilateral parietal regions significantly increased the discrimination sensitivity compared to a sham stimulation condition. More recently, Vulić et al. (2021) compared the effects of two types of anodal tDCS over the left PPC on cued recall of face-word associations, i.e., standard tDCS and frequency-modulated tDCS oscillating in theta rhythm (5 Hz). This study shows that both types of parietal stimulation led to enhanced associative memory performance and highlights the potential of oscillatory tDCS protocols for memory enhancement. In this context, an interesting method to investigate cortical-hippocampal structures is to target with TMS the cortical sites based on their high fMRI connectivity with anatomically defined hippocampal locations. Wang et al. (2014) applied off-line trains of high-frequency (20 Hz) rTMS to a left parietal site for 5 days and found an improvement of associative memory of previously learned items. A role of the left parietal cortex using this method was confirmed by further studies using high-frequency rTMS trains (Nilakantan et al., 2017; Kim et al., 2018; Tambini et al., 2018). A recent study using off-line rTMS over the left parietal cortex at different frequencies reported facilitation of memory retrieval following the application of continuous theta burst stimulation (cTBS) associated with corresponding increases in fMRI connectivity between the hippocampus and cortical areas (Hermiller et al., 2019).

In contrast, other studies aimed at investigating the role of low-frequency off-line rTMS of frontal and parietal regions of the cortical-hippocampal network in recognition memory. Turriziani et al. (2012) first reported facilitation of non-verbal recognition memory when 1 Hz rTMS trains were applied over the right DLPFC before the recognition but not before the encoding phase in both healthy subjects and patients with mild cognitive impairment. Interestingly, these authors found that the application of high-frequency rTMS to the right DLPFC, using the intermittent Theta Burst Stimulation (iTBS) protocol, worsened recognition memory performance. The same pattern of results was then replicated using cathodal tDCS in healthy subjects (Smirni et al., 2015).

Here we investigated whether 1 Hz rTMS applied to the posterior parietal cortex (PPC) modulates recognition memory tasks depending on the hemisphere (i.e., right vs. left) and on the timing of the stimulation (i.e., encoding vs. retrieval) of the task.

## Materials and Methods

To investigate the role of PPC in the non-verbal recognition memory we conducted two rTMS experiments employing a cross-over sham-controlled design with stimulation condition (sham vs. active rTMS) as within-subject factor and side (left vs. right PPC) as between-subjects factor.

In experiment 1, subjects received sham and active rTMS over the left and the right PPC before the encoding phase.

**FIGURE 1:**
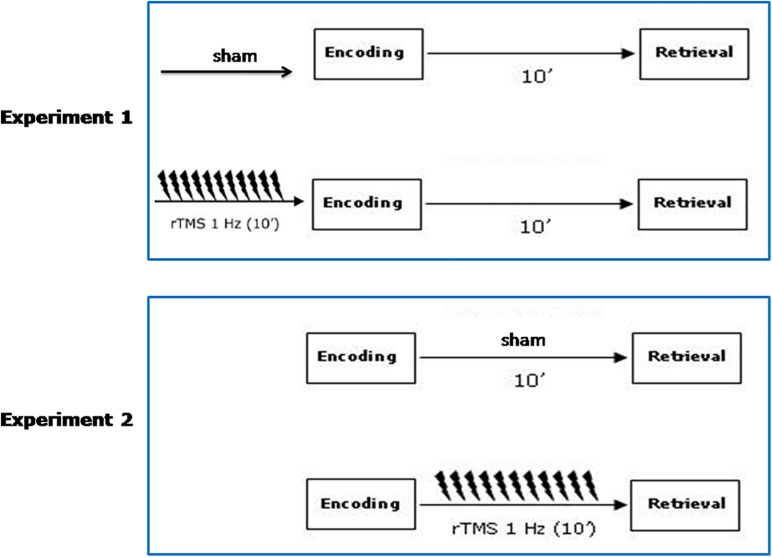
Schematic diagram of the experimental procedure. Participants received sham and active rTMS, in two counterbalanced separate sessions on the same day. A 6 h washout interval was applied. In Experiment 1, sham/active rTMS over the left and the right PPC was given before the encoding phase. In Experiment 2, sham/active rTMS over the left and the right PPC was given before the retrieval phase.

In experiment 2, subjects received sham and active rTMS over the left and the right PPC before the retrieval phase. See Figure 1 for a schematic diagram of the experimental procedure.

### Participants

We recruited 40 right-handed participants (5 males, 35 females; mean age: 22 ± 4 years; education level: 14 ± 1 years) out of which 20 were enrolled in Experiment 1 (4 males, 16 females; mean age: 22 ± 3 years; education level: 14 ± 2 years) and 20 in Experiment 2 (1 male, 19 females; mean age: 22 ± 6 years; education level: 14 ± 1 years) and half of each was assigned to either left or right PPC group (*N* = 10) receiving sham and active rTMS. All the participants were native Italian speakers, naive to the purposes of the study. Inclusion criteria were: normal or corrected to normal vision, no previous history of neurological or psychiatric problems. Participants were free of any medication and they were screened for exclusion criteria for TMS (Rossi et al., 2011).

The study was conducted in accordance with the principles of the Declaration of Helsinki and was approved by the local Bioethics Committee of the University of Palermo (n° 26/2020). Written informed consent was obtained from all subjects.

### Experiment 1: 1 Hz Repetitive Transcranial Magnetic Stimulation of the Posterior Parietal Cortex Before the Encoding Phase

The aim of the first experiment was to investigate the effect of the left and right PPC before the memory encoding.

### Materials

The materials used have been employed in previous studies where they have been described in detail (Turriziani et al., 2012, 2019; Smirni et al., 2015). The experimental task is an Italian version (Smirni et al., 2010) of the Recognition Memory Test. The stimuli used were unknown faces. To apply different stimuli in sham and rTMS sessions, we used two computerized parallel forms of stimuli: the first (Form A) was the Faces Recognition Test from Smirni et al. (2010); the second (Form B) was a parallel form of the same test (Smirni et al., 2018). In both Forms, there was a study phase, we termed this “encoding phase,” followed by a test phase, we termed this “retrieval phase.”

In the encoding phase stimuli were 30 faces The faces were black and white photographs of Caucasian women, approximately 25 years old, with Italian physiognomic characteristics, neutral expression, and no obvious distinguishing features.

### Procedure

Procedures were identical in both forms (A and B). Sham and active rTMS were given before the encoding phase, in two separate sessions on the same day, separated by a 6 h delay. In each condition (sham or active rTMS) the subjects were administered either the Form A or the Form B of recognition tests. Administration of these two tasks was counterbalanced across subjects, for Form A and Form B and for sham and active rTMS conditions.

In the study phase, stimuli were presented individually in the center of a 15” computer screen over a white background for 2,000 ms. The stimuli were preceded by a fixation point lasting 500 ms. The inter-stimulus interval (ISI) was 3,500 ms.

Participants were instructed to judge whether the stimulus presented in the study phase was “pleasant” or “unpleasant” to focus subjects’ attention in stimulus encoding. Participants responded by pressing one of two designated keys on the keyboard.

Therefore, in the first session (sham or active rTMS) the task requires incidental encoding whereas in the second session we cannot exclude that subjects had the previous experience of needing to remember faces for subsequent recognition.

The recognition phase was administered after a delay interval of 10 min. During this interval, in all experimental conditions, the examiner engaged the subjects in an everyday conversation focusing on various life topics.

In the recognition phase, a three alternative forced-choice recognition memory task was administered. Thirty stimulus triplets were presented. In each triplet, the target was presented with two other similar distractors, vertically arranged. The target was presented in a balanced order either in the upper, lower, or middle quadrant of the screen.

The distractors were two faces with physiognomic characteristics similar to the target. This similarity was previously established in a pilot study, in which participants were asked to judge the face similarity based on hair and color configuration, eyes color and shape, nose and mouth shape.

The recognition trial began with a fixation point of 500 ms, followed by the presentation of the triplets (target and two distractors) for 2,000 ms, followed by the presentation of followed by a blank screen for 3,000 ms. The ISI was 3,500 ms. Subjects were asked to recognize the previously presented stimuli by pressing one of three designated keys on the keyboard using the right index finger from the onset of the test stimuli until 3,000 ms after its disappearance. If unsure they were asked to guess.

Responses were measured in terms of accuracy and reaction times (RTs). Accuracy was considered as the number of correct targets that participants were able to identify in the three forced-choice recognition memory test. The RTs were considered as the time interval from the onset of the test stimuli to the subject’s response.

### Repetitive Transcranial Magnetic Stimulation Protocol

A MagStim Super Rapid magnetic stimulator (Whitland, United Kingdom) was used. The stimulator was connected to a focal figure-of-eight coil (diameter: 70 millimeters). In each condition (sham and active rTMS), the center of the coil wings was positioned at a position on the scalp corresponding to P3 and P4 sites of the 10–20 EEG system as well-established targets for PPC stimulation (e.g., see Mangano et al., 2015; Bjekić et al., 2019). In the rTMS condition, the figure-of-eight coil was applied tangentially on the target scalp site, with the handle pointing posteriorly, to induce a current with posterior-to-anterior direction in the underlying brain areas. In the sham condition, rTMS was applied, with the coil held close to the PPC but angled away. The same intensity and timing of rTMS were used for sham stimulation. In this case, the coil was still centered on P3 and P4 sites, but it was held perpendicular to the scalp surface, so that scalp contact and discharging noise were quite similar to those for active stimulation, but the induced magnetic field did not activate cortical neurons. The coil was maintained on the target scalp site with the same orientation across sessions using a mechanical arm while the participants quietly sat.

For each scalp site, an rTMS train of 10 min duration and 1 Hz frequency (600 stimuli) was applied at an intensity of 90% of the resting motor threshold (MT) which was defined as the minimal TMS intensity capable of inducing a reliable muscle twitch in the contralateral hand on 50% of trials within a sequence of ten consecutive trials (Rossini et al., 1994).

MT was determined on the same hemisphere of the stimulated left or right PPC.

There were not interhemispheric differences in MT values between the left and the right hemisphere either in experiment 1 (MT left group: 55 ± 8%; MT right group: 55 ± 7%; *p* = 0.97) or in experiment 2 (MT left group: 56 ± 5%; MT right group: 58 ± 7%; *p* = 0.35).

**FIGURE 2:**
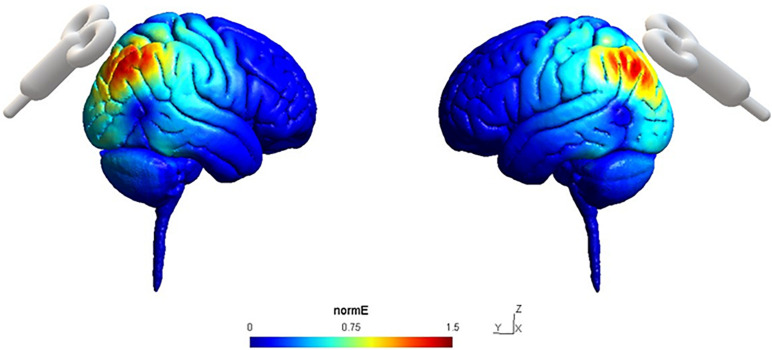
Simulation of normalized Electric field (Norm*E*) distribution in the sites of stimulation P4 (left) and P3 (right) on a sagittal section using SimNIBS 3.0. Norm*E* intensity is color-coded from 0 to 1.5 mm/mV.

We also conducted the simulations of the electric field patterns that rTMS would evocate in cerebral cortices of the subjects using SimNIBS 3.0 (Saturnino et al., 2019; Figure 2).

### Experiment 2: 1 Hz Repetitive Transcranial Magnetic Stimulation of the Posterior Parietal Cortex Before the Retrieval Phase

The aim of the second experiment was to investigate the effect of the left and right PPC before the memory retrieval.

The same stimuli, procedures, and rTMS protocol as those administered in experiment 1 were used, with the exception that subjects received sham and active rTMS to PPC before the retrieval phase.

### Data Analysis

We calculated the accuracy, that is the percentage of correctly recognized items in the three forced-choice recognition memory test, and the averaged reaction times (RTs), defined as the time interval from the onset of the test stimuli to the correct button presses.

Data were analyzed with separate ANOVAs on the accuracy and the averaged RTs, with Side (left vs. right hemisphere) as between-subjects factor and Condition (sham, active rTMS) as within-subjects factor with simple planned contrasts between sham and active rTMS conditions and between left and right group in each conditions (left rTMS vs. right active rTMS; left sham vs. right sham). Value of *p* < 0.05 was considered as significant.

Finally, we calculated the sensitivity index as a measure of participants ability to discriminate between signal and noise dividing the number of correct responses by the total number of trials (Stanislaw and Todorov, 1999). A value of 0.3 was considered as indicating chance performance.

## Results

### Experiment 1: 1 Hz Repetitive Transcranial Magnetic Stimulation of the Posterior Parietal Cortex Before the Encoding Phase

#### Accuracy

Overall participants were accurate choosing the correct answer more often than predicted by chance. The mean percentage of correct responses for the sham and active rTMS conditions, respectively, were: 77 (*SD* = 6) and 76 (*SD* = 12) for the left group; 76 (*SD* = 9) and 77 (*SD* = 11) for the right group.

**FIGURE 3:**
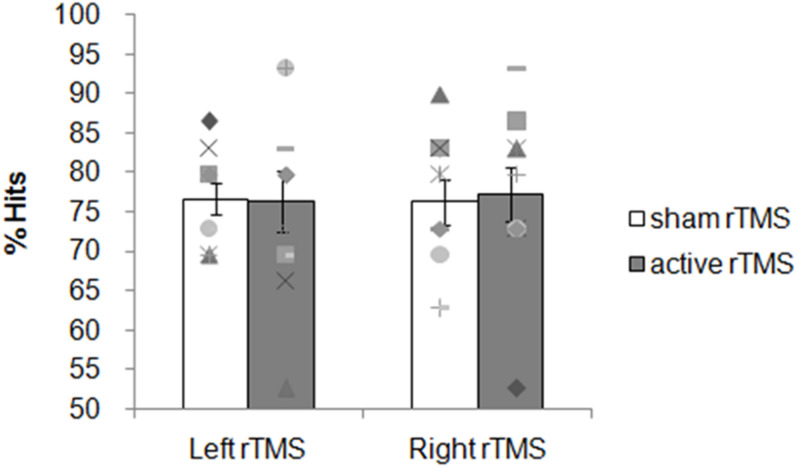
Experiment 1: 1 Hz rTMS of the PCC before the encoding phase. Mean percentage of subjects’ correct responses and individual performances. Each symbol represents one participant; bar plots represent the average over participants, and error bars indicate standard error.

The ANOVA on the accuracy showed no significant main effects for factors Side [F(1, 18) = 0.01; *p* = 0.9159], Condition [*F*(1, 18) = 0.01; *p* = 0.9163] nor Side × Condition interaction [*F*(1, 18) = 0.05; *p* = 0.8336; Figure 3]. Planned contrasts revealed no significant difference between sham and active rTMS [*F*(1, 19) = 0.01; *p* = 0.91]. The contrast between left and right sham rTMS was not significant [*F*(1, 18) = 0.009; *p* = 0.93] as well as the contrast between right active rTMS and left active rTMS [*F*(1, 18) = 0.03; *p* = 0.84].

#### Reaction Times

The averaged RTs for the sham and active rTMS conditions, respectively, were: 1,849 ms (*SD* = 154) and 1,971 ms (*SD* = 154) for the left group; 1,925 ms (*SD* = 283) and 1,913 ms (*SD* = 324) for the right group. No significant main effects on the averaged RTs for the factors Side [*F*(1, 18) = 0.01; *p* = 0.9225], Condition [*F*(1, 18) = 1.47; *p* = 0.2408], nor Side × Condition interaction [*F*(1, 18) = 2.12; *p* = 0.1630] were found. Planned contrasts revealed no significant difference between sham and active rTMS [*F*(1, 19) = 1.39; *p* = 0.25]. The contrast between left and right sham rTMS conditions was not significant [*F*(1, 18) = 0.57; *p* = 0.45] as well as the contrast between right active rTMS and left active rTMS [*F*(1, 18) = 0.24; *p* = 0.62]. These results indicate that active rTMS over the left and right PPC at encoding did not modulate the performance on the non-verbal recognition memory task.

### Experiment 2: 1 Hz Repetitive Transcranial Magnetic Stimulation of the Posterior Parietal Cortex Before the Retrieval Phase

#### Accuracy

**FIGURE 4:**
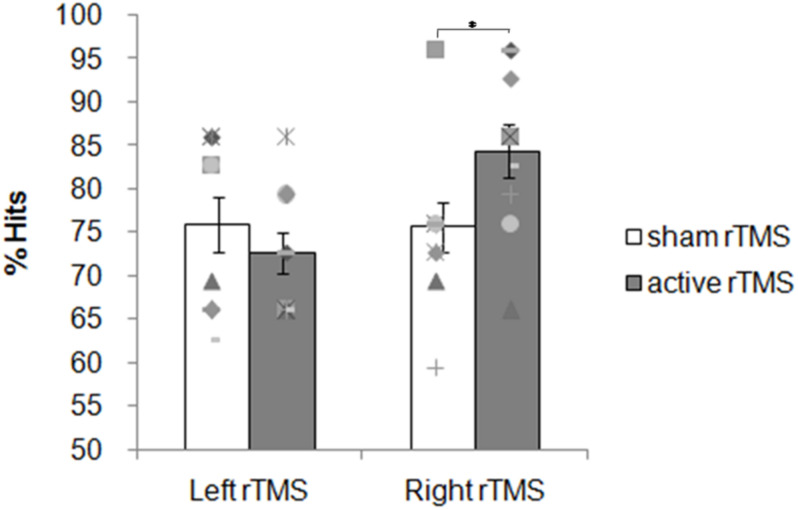
Experiment 2: 1 Hz rTMS of the PPC before the retrieval phase. Mean percentage of subjects’ correct responses and individual performances. Each symbol represents one participant; bar plots represent the average over participants, and error bars indicate standard error.

Overall participants were accurate choosing the correct answer more often than predicted by chance. The mean percentage of correct responses for the sham and active rTMS conditions, respectively, were: 76 (*SD* = 10) and 72 (*SD* = 7) for the left group; 76 (*SD* = 9) and 84 (*SD* = 10) for the right group. The ANOVA on the accuracy showed no significant main effects for the factors Side [*F*(1, 18) = 3.02; *p* = 0.09] and Condition [*F*(1, 18) = 1.21; *p* = 0.28]. A significant interaction Side × Condition was found [*F*(1, 18) = 6.13; *p* = 0.02; η*_*p*_*^2^ = 0.25; Figure 4). The contrast between sham and active rTMS was not significant [*F*(1, 19) = 0.95; *p* = 0.34]. Planned contrasts revealed that right active rTMS significantly improved subjects’ accuracy when compared with left active rTMS [*F*(1, 18) = 9.21; *p* = 0.007; η*_*p*_*^2^ = 0.33] while there was no significant difference between left and right sham rTMS conditions [*F*(1, 18) = 0.006, *p* = 0.93; η*_*p*_*^2^ = 0.0003].

#### Reaction Times

The averaged RTs for the sham and active rTMS conditions, respectively, were: 1,813 ms (*SD* = 296) and 1,794 ms (*SD* = 304) for the left group; 1,956 ms (*SD* = 253) and 1,881 ms (*SD* = 367) for the right group. The ANOVA on the averaged RTs showed no significant main effects for any factor Side [*F*(1, 18) = 0.74; *p* = 0.40], Condition [*F*(1, 18) = 1.87; *p* = 0.18] nor Side × Condition interaction [*F*(1, 18) = 0.68; *p* = 0.41]. Planned contrast revealed no significant difference between sham and active rTMS [*F*(1, 19) = 1.89; *p* = 0.18]. The contrast between left and right sham rTMS conditions was not significant [*F*(1, 18) = 1.35, *p* = 0.26] as well as the contrast between right active rTMS and left active rTMS [*F*(1, 18) = 0.32; *p* = 0.57]. These results indicate that active rTMS over the right, but not left, PPC before the retrieval phase significantly improves non-verbal recognition memory performance without any significant modulation of speed of response.

## Discussion

The main results of the present study show that 1 Hz rTMS of the right PPC selectively enhanced item-recognition accuracy when it was applied before the retrieval phase, whereas no effects were found when rTMS was administered over the left PPC or before the encoding phase over either the right or the left PPC.

These results are in accord with neuroimaging findings documenting the role of PPC in recognition memory (Cabeza and Nyberg, 2000; Rugg et al., 2002; Wagner et al., 2005). Moreover, the process-specific effect of TMS related to the retrieval but not to the encoding phase of a recognition memory task is in line with previous studies reporting involvement of the PPC in the retrieval phase of long term memory (e.g., Manenti et al., 2010; Sestieri et al., 2013, 2017).

A recent meta-analysis of the TMS effects for neuromodulation of episodic memory (Yeh and Rose, 2019), has not documented clear-cut different effects of rTMS when stimulating the left or the right hemisphere during either encoding or retrieval. The present results add new findings to this debate, by showing modulation of episodic memory linked to both hemispheric lateralization and timing of the stimulation, which may be important for future research.

Concerning the hemispheric lateralization, as discussed in the Introduction, previous findings suggest that the retrieval-related activity may be stronger in the left hemisphere (but see Koen et al., 2018 for a revision of the role of the left angular gyrus in encoding). Our data suggest that 1-Hz rTMS delivered to the left PPC does not affect facial memory retrieval.

Indeed, the question of a possible hemispheric specialization of PPC in memory retrieval is still open. Lesion studies that found memory-related deficits following parietal lesions recruited mostly patients with bilateral lesions (Berryhill et al., 2007; Drowos et al., 2010; Simons et al., 2010). Neuroimaging studies reported left-lateralized parietal activations although the degree of fMRI lateralization is not the same across studies. Consequently, most TMS studies delivered rTMS stimulation only to left PPC regions (Sestieri et al., 2013; Wang et al., 2014; Nilakantan et al., 2017; Kim et al., 2018; Tambini et al., 2018). Other TMS studies that stimulated both left and right PPC employed verbal stimuli and found memory-related effects during left PPC rTMS (Manenti et al., 2010). On the other hand, in the present study, we used non-verbal stimuli, and thus we cannot exclude the influence of modality specific memory effects on hemispheric lateralization during retrieval. With respect to the timing of the stimulation, we found specific TMS effects related to the retrieval phase. It may be interesting to note that most TMS studies in the literature apply forms of intentional encoding, while in the present study we used incidental encoding at least in our first session. According to the level of processing framework (Craik and Lockhart, 1972), deep (i.e., semantic) compared to shallow (i.e., perceptual and incidental) encoding benefits later retrieval (Galli, 2014). Moreover, dorsal vs. ventral PPC areas make opposite contributions during encoding (Uncapher and Wagner, 2009). Since we stimulated a single region of the PPC, it may be that the lack of rTMS effects on encoding in the present study is related to both the nature of encoding (incidental rather than intentional) and the stimulated parietal site.

Results of the present study show an improvement of the performance despite the use of a neuromodulation paradigm that is usually characterized as inhibitory for the stimulated cortex, at least at the motor cortical level (Pascual-Leone et al., 1998; Thickbroom, 2007). One may therefore think that the effects of TMS may differ according to the targeted region (e.g., motor cortex vs. prefrontal/parietal cortex). Related to this, our results are in line with a previous study documenting an improvement on the same recognition memory task after 1 Hz rTMS of the right DLPFC in healthy subjects and in patients with mild cognitive impairment (Turriziani et al., 2012) and, more recently, in patients with Alzheimer’s disease (Turriziani et al., 2019). This result is also in accord with the literature on TMS effects on episodic memory, showing larger facilitatory effects of 1 Hz rTMS compared with other stimulation frequencies (Yeh and Rose, 2019).

We adopted an off-line low-frequency rTMS protocol that is regarded to induce plastic effects even at brain sites distant from those stimulated. As a consequence, one of the explanations proposed to account for this memory facilitation after 1 Hz rTMS inhibition of the right DLPFC or PPC, pointed to the modulation of excitability of the target region as well as of anatomically and functionally interconnected brain structures such as the middle temporal lobe (e.g., Simons and Spiers, 2003; Wagner et al., 2005; Vilberg and Rugg, 2008).

The present results could also be consistent with the Attention to Memory model (Cabeza et al., 2008; Ciaramelli et al., 2008, 2020), according to which activity of the dorsal parietal cortex maintains retrieval goals, which modulate memory-related activity in the medial temporal lobe. We suggest that the improvement of recognition memory after 1 Hz rTMS of the right PPC could be correlated with the high functional connectivity between the lateral parietal cortex and the hippocampus (Kahn et al., 2008), likely mediated by lateral parietal projections to retrosplenial and parahippocampal cortex (Mesulam et al., 1977; Cavada and Goldman-Rakic, 1989a,b; Vincent et al., 2006, 2008).

Our results are in line with those of a recent study, testing the relationships between memory retrieval and hippocampal fMRI connectivity using rTMS at different frequencies (Hermiller et al., 2019). The authors reported facilitation of memory retrieval following the application of continuous theta burst stimulation (cTBS) over the left parietal cortex. Individual differences in retrieval improvements following cTBS were associated with corresponding increases in fMRI connectivity between the hippocampus and cortical areas, consistent with the idea that the influence of TMS of the parietal cortex may be related to the ability to distally target the hippocampus and contiguous regions of the mesial temporal lobe.

Facilitation of recognition memory following 1 Hz rTMS of the right hemisphere is not at odds with the findings of memory retrieval facilitation using high-frequency rTMS of the left hemisphere reported in previous rTMS studies. In both cases, i.e., high-frequency rTMS of the left parietal cortex or low-frequency rTMS of the right parietal cortex would facilitate guided retrieval of relevant memory representations via facilitation of network connectivity mediated by transcallosal interactions.

The present study has some limitations to be considered when interpreting these results. The first one is the small numerosity of the sample. *Post hoc* power analyses conducted using G^∗^Power 3 software (Faul et al., 2007) revealed that the observed power for experiment 1 was 0.31 and 0.93 for the accuracy and RTs results, respectively; while the observed power for experiment 2 was 0.99 and 0.77 for the accuracy and RTs results, respectively, suggesting that the study was underpowered. Another limit is that we targeted a single region of the parietal cortex. Since several parietal sub-regions probably play distinct roles during memory retrieval (Hutchinson et al., 2014), the present results cannot test different accounts, based on the role of attention (Cabeza et al., 2008; O’Connor et al., 2010), event representation (Vilberg and Rugg, 2008; Shimamura, 2011), or decision making (Donaldson et al., 2010) processes.

Another limit of the study is in the sham/control procedure. In fact, tilting the coil away from the target scalp site does not induce the same kind of somatosensory stimulation as active stimulation, a problem existing in many TMS studies even when a sham coil is used.

In summary, we have demonstrated that 1 Hz rTMS of the right PPC selectively enhanced recognition memory when it was applied before the retrieval phase. Further research employing different memory stimuli and targetting different parietal sites in a representative sample, will be required.

## Data Availability Statement

The raw data supporting the conclusions of this article will be made available by the authors, without undue reservation.

## Ethics Statement

The studies involving human participants were reviewed and approved by Bioethics Committee of the University of Palermo. The patients/participants provided their written informed consent to participate in this study.

## Author Contributions

MO, DS, and PT designed the research. GRM and DS collected the data. GRM, VT, and PT analysed and discussed the data. GRM, MO, and PT wrote the manuscript.

## Conflict of Interest

The authors declare that the research was conducted in the absence of any commercial or financial relationships that could be construed as a potential conflict of interest.

## Publisher’s Note

All claims expressed in this article are solely those of the authors and do not necessarily represent those of their affiliated organizations, or those of the publisher, the editors and the reviewers. Any product that may be evaluated in this article, or claim that may be made by its manufacturer, is not guaranteed or endorsed by the publisher.

## References

[B1] Berryhill M. E. Phuong L. Picasso L. Cabeza R. Olson I. R. (2007). Parietal lobe and episodic memory: bilateral damage causes impaired free recall of autobiographical memory. *J. Neurosci.* 27 14415–14423. 10.1523/JNEUROSCI.4163-07.2007 18160649 PMC6673454

[B2] Bjekić J. Čolić M. V. Živanović M. Milanović S. D. Filipović S. R. (2019). Transcranial direct current stimulation (tDCS) over parietal cortex improves associative memory. *Neurobiol. Learn. Mem.* 157 114–120. 10.1016/j.nlm.2018.12.007 30553021

[B3] Cabeza R. Ciaramelli E. Olson I. R. Moscovitch M. (2008). The parietal cortex and episodic memory: an attentional account. *Nat. Rev.Neurosci.* 9 613–625. 10.1038/nrn2459 18641668 PMC2692883

[B4] Cabeza R. Nyberg L. (2000). Neural bases of learning and memory: Functional neuroimaging evidence. *Curr. Opin. Neurol.* 13 415–421. 10.1097/00019052-200008000-00008 10970058

[B5] Cavada C. Goldman-Rakic P. S. (1989a). Posterior parietal cortex in rhesus monkey: I. Parcellation of areas based on distinctive limbic and sensory cortico cortical connections. *J. Comp.Neurol.* 287 393–421. 10.1002/cne.902870402 2477405

[B6] Cavada C. Goldman-Rakic P. S. (1989b). Posterior parietal cortex in rhesus monkey: II. evidence for segregated corticocortical networks linking sensory and limbic areas with the frontal lobe. *J. Comp.Neurol.* 287 422–445. 10.1002/cne.902870403 2477406

[B7] Ciaramelli E. Burianová H. Vallesi A. Cabeza R. Moscovitch M. (2020). Functional Interplay between posterior parietal cortex and hippocampus during detection of memory targets and non-targets. *Front. Neurosci.* 14:563768. 10.3389/fnins.2020.563768 33224020 PMC7670044

[B8] Ciaramelli E. Grady C. L. Moscovitch M. (2008). Top-down and bottom-up attention to memory: a hypothesis (AtoM) on the role of the posterior parietal cortex in memory retrieval. *Neuropsychologia* 46 1828–1851. 10.1016/j.neuropsychologia.2008.03.022 18471837

[B9] Craik F. I. M. Lockhart R. S. (1972). Levels of processing. A framework for memory research. *J. Verb. Learn. Verb. Behav.* 11 671–684. 10.1016/S0022-5371(72)80001-X

[B10] Donaldson D. I. Wheeler M. E. Petersen S. E. (2010). Remember the source: dissociating frontal and parietal contributions to episodic memory. *J. Cogn. Neurosci.* 22 377–391. 10.1162/jocn.2009.21242 19400677

[B11] Drowos D. B. Berryhill M. André J. M. Olson I. R. (2010). True memory, false memory, and subjective recollection deficits after focal parietal lobe lesions. *Neuropsychology* 24 465–475. 10.1037/a0018902 20604621 PMC2917642

[B12] Faul F. Erdfelder E. Lang A. G. Buchner A. (2007). G^∗^Power 3: a flexible statistical power analysis program for the social, behavioral, and biomedical sciences. *Behav. Res. Methods.* 39 175–191. 10.3758/bf03193146 17695343

[B13] Gagnon G. Blanchet S. Grondin S. Schneider C. (2010). Paired-pulse transcranial magnetic stimulation over the dorsolateral prefrontal cortex interferes with episodic encoding and retrieval for both verbal and non-verbal materials. *Brain Res.* 1344 148–158. 10.1016/j.brainres.2010.04.041 20462501

[B14] Galli G. (2014). What makes deeply encoded items memorable? Insights into the levels of processing framework from neuroimaging and neuromodulation. *Front. Psychiatry* 5:61. 10.3389/fpsyt.2014.00061 24904444 PMC4035598

[B15] Hermiller M. S. VanHaerents S. Raij T. Voss J. L. (2019). Frequency-specific non invasive modulation of memory retrieval and its relationship with hippocampal network connectivity. *Hippocampus* 29 595–609. 10.1002/hipo.23054 30447076 PMC6525080

[B16] Hutchinson J. B. Uncapher M. R. Weiner K. S. Bressler D. W. Silver M. A. Preston A. R. (2014). Functional heterogeneity in posterior parietal cortex across attention and episodic memory retrieval. *Cereb. Cortex* 24 49–66. 10.1093/cercor/bhs278 23019246 PMC3862264

[B17] Kahn I. Andrews-Hanna J. R. Vincent J. L. Snyder A. Z. Buckner R. L. (2008). Distinct cortical anatomy linked to subregions of the medialtemporal lobe revealed by intrinsic functional connectivity. *J. Neurophysiol.* 100 129–139. 10.1152/jn.00077.2008 18385483 PMC2493488

[B18] Kim K. Ekstrom A. D. Tandon N. (2016). A network approach for modulating memory processes via direct and indirect brain stimulation: toward a causal approach for the neural basis of memory. *Neurobiol. Learn. Mem.* 134(Pt A) 162–177. 10.1016/j.nlm.2016.04.001 27066987

[B19] Kim S. Nilakantan A. S. Hermiller M. S. Palumbo R. T. VanHaerents S. Voss J. L. (2018). Selective and coherent activity increases due to stimulation indicate functional distinctions between episodic memory networks. *Sci. Adv.* 4:eaar2768. 10.1126/sciadv.aar2768 30140737 PMC6105230

[B20] Koen J. D. Thakral P. P. Rugg M. D. (2018). Transcranial magnetic stimulation of the left angular gyrus during encoding does not impair associative memory performance. *Cogn. Neurosci.* 9 127–138. 10.1080/17588928.2018.1484723 29870300 PMC6185791

[B21] Manenti R. Tettamanti M. Cotelli M. Miniussi C. Cappa S. F. (2010). The neural bases of word encoding and retrieval: a fMRI-guided transcranial magnetic stimulation study. *Brain Topogr.* 22 318–332. 10.1007/s10548-009-0126-1 20012682

[B22] Mangano G. R. Oliveri M. Turriziani P. Smirni D. Zhaoping L. Cipolotti L. (2015). Repetitive transcranial magnetic stimulation over the left parietal cortex facilitates visual search for a letter among its mirror images. *Neuropsychologia* 70 196–205. 10.1016/j.neuropsychologia.2015.03.002 25744867

[B23] McClelland J. J. L. McNaughton B. B. L. Bruce L. O’Reilly R. C. (1995). Why there are complementary learning systems in the hippocampus and neocortex: insights from the successes and failures of connectionist models of learning and memory. *Psychol. Rev.* 102 419–457. 10.1037/0033-295X.102.3.419 7624455

[B24] Mesulam M. M. Van Hoesen G. W. Pandya D. N. Geschwind N. (1977). Limbic and sensory connections of the inferior parietal lobule (area PG) in the rhesus monkey: a study with a new method for horseradish peroxidase histochemistry. *Brain Res.* 136 393–414. 10.1016/0006-8993(77)90066-x 411543

[B25] Nadel L. Samsonovich A. Ryan L. Moscovitch M. (2000). Multiple trace theory of human memory: computational, neuroimaging, and neuropsychological results. *Hippocampus* 10 352–368. 10.1002/1098-1063200010:4<352::AID-HIPO2>3.0.CO;2-D 10985275

[B26] Nilakantan A. S. Bridge D. J. Gagnon E. P. VanHaerents S. A. Voss J. L. (2017). Stimulation of the posterior cortical-hippocampal network enhances precision of memory recollection. *Curr. Biol.* 27 465–470. 10.1016/j.cub.2016.12.042 28111154 PMC5302852

[B27] O’Connor A. R. Han S. Dobbins I. G. (2010). The inferior parietal lobule and recognition memory: expectancy violation or successful retrieval? *J. Neurosci.* 30 2924–2934. 10.1523/JNEUROSCI.4225-09.2010 20181590 PMC2844718

[B28] Pascual-Leone A. Tormos J. M. Keenan J. Tarazona F. Cañete C. Catalá M. D. (1998). Study and modulation of human cortical excitability with transcranial magnetic stimulation. *J. Clin. Neurophysiol.* 15 333–343. 10.1097/00004691-199807000-00005 9736467

[B29] Pisoni A. Turi Z. Raithel A. Ambrus G. G. Alekseichuk I. Schacht A. (2015). Separating recognition processes of declarative memory via anodal tDCS: boosting old item recognition by temporal and new item detection by parietal stimulation. *PLoS One* 10:e0123085. 10.1371/journal.pone.0123085 25816233 PMC4376742

[B30] Rossi S. Cappa S. F. Babiloni C. Pasqualetti P. Miniussi C. Carducci F. (2001). Prefrontal [correction of Prefontal] cortex in long-term memory: an “interference” approach using magnetic stimulation. *Nat. Neurosci.* 2001;4 948–952. 10.1038/nn0901-948. *Erratum Nat. Neurosci.* 5:1017. 11528428

[B31] Rossi S. Hallett M. Rossini P. M. Pascual-Leone A. (2011). Screening questionnaire before TMS: an update. *Clin. Neurophysiol.* 122:1686. 10.1016/j.clinph.2010.12.037 21227747

[B32] Rossi S. Pasqualetti P. Zito G. Vecchio F. Cappa S. F. Miniussi C. (2006). Prefrontal and parietal cortex in human episodic memory: an interference study by repetitive transcranial magnetic stimulation. *Eur. J. Neurosci.* 23 793–800. 10.1111/j.1460-9568.2006.04600.x 16487159

[B33] Rossini P. M. Barker A. T. Berardelli A. Caramia M. D. Caruso G. Cracco R. Q. (1994). Non-invasive electrical and magnetic stimulation of the brain, spinal cord and roots: basic principles and procedures for routine clinical application. Report of an IFCN committee. *Electroencephalogr. Clin. Neurophysiol.* 91 79–92. 10.1016/0013-4694(94)90029-9 7519144

[B34] Rugg M. D. Otten L. J. Henson R. N. A. (2002). The neural basis of episodic memory: evidence from functional neuroimaging. *Philos. Trans. R. Soc. B Biol. Sci.* 357 1097–1110. 10.1098/rstb.2002.1102 12217177 PMC1693015

[B35] Sandrini M. Cappa S. F. Rossi S. Rossini P. M. Miniussi C. (2003). The role of prefrontal cortex in verbal episodic memory: rTMS evidence. *J. Cogn. Neurosci.* 15 855–861. 10.1162/089892903322370771 14511538

[B36] Saturnino G. B. Madsen K. H. Thielscher A. (2019). Electric field simulations for transcranial brain stimulation using FEM: an efficient implementation and error analysis. *J. Neural. Eng.* 16:066032. 10.1088/1741-2552/ab41ba 31487695

[B37] Sestieri C. Capotosto P. Tosoni A. Luca Romani G. Corbetta M. (2013). Interference with episodic memory retrieval following transcranial stimulation of the inferior but not the superior parietal lobule. *Neuropsychologia* 51 900–906. 10.1016/j.neuropsychologia.2013.01.023 23391557

[B38] Sestieri C. Shulman G. L. Corbetta M. (2017). The contribution of the human posterior parietal cortex to episodic memory. *Nat. Rev. Neurosci.* 18 183–192. 10.1038/nrn.2017.6 28209980 PMC5682023

[B39] Shimamura A. P. (2011). Episodic retrieval and the cortical binding of relational activity. *Cogn. Affect. Behav. Neurosci.* 11 277–291. 10.3758/s13415-011-0031-4 21638193

[B40] Simons J. S. Peers P. V. Mazuz Y. S. Berryhill M. E. Olson I. R. (2010). Dissociation between memory accuracy and memory confidence following bilateral parietal lesions. *Cereb. Cortex* 20 479–485. 10.1093/cercor/bhp116 19542474 PMC2803741

[B41] Simons J. S. Spiers H. J. (2003). Prefrontal and medial temporal lobe interactions in long-term memory. *Nat. Rev. Neurosci.* 4 637–648. 10.1038/nrn1178 12894239

[B42] Smirni D. Smirni P. Di Martino G. Cipolotti L. Oliveri M. Turriziani P. (2018). Standardization and validation of a parallel form of the verbal and non-verbal recognition memory test in an Italian population sample. *Neurol. Sci.* 39 1391–1399. 10.1007/s10072-018-3433-z 29728938

[B43] Smirni D. Turriziani P. Mangano G. R. Cipolotti L. Oliveri M. (2015). Modulating memory performance in healthy subjects with transcranial direct current stimulation over the right dorsolateral prefrontal cortex. *PLoS One* 10:e0144838. 10.1371/journal.pone.0144838 26679936 PMC4682999

[B44] Smirni D. Turriziani P. Oliveri M. Smirni P. Cipolotti L. (2010). Standardizzazione di tre nuovi test di memoria di riconoscimento verbale e non verbale: uno studio preliminare. *G. Ital. Psicol.* 1 227–245.

[B45] Stanislaw H. Todorov N. (1999). Calculation of signal detection theory measures. *Behav. Res. Methods Instrum. Comput.* 31 137–149. 10.3758/bf03207704 10495845

[B46] Tambini A. Nee D. E. D’Esposito M. (2018). Hippocampal-targeted theta-burst stimulation enhances associative memory formation. *J. Cogn. Neurosci.* 30 1452–1472. 10.1162/jocn_a_01300 29916791 PMC7467684

[B47] Teyler T. J. Di Scenna P. (1986). The hippocampal memory indexing theory. *Behav. Neurosci.* 100 147–154. 10.1037//0735-7044.100.2.147 3008780

[B48] Thickbroom G. W. (2007). Transcranial magnetic stimulation and synaptic plasticity: experimental framework and human models. *Exp. Brain Res.* 180 583–593. 10.1007/s00221-007-0991-3 17562028

[B49] Turriziani P. Smirni D. Mangano G. R. Zappalà G. Giustiniani A. Cipolotti L. (2019). Low-frequency repetitive transcranial magnetic stimulation of the right dorsolateral prefrontal cortex enhances recognition memory in Alzheimer’s disease. *J. Alzheimers Dis.* 72 613–622. 10.3233/JAD-190888 31609693

[B50] Turriziani P. Smirni D. Zappalà G. Mangano G. R. Oliveri M. Cipolotti L. (2012). Enhancing memory performance with rTMS in healthy subjects and individuals with mild cognitive impairment: the role of the right dorsolateral prefrontal cortex. *Front. Hum. Neurosci.* 10:62. 10.3389/fnhum.2012.00062 22514525 PMC3322484

[B51] Uncapher M. Wagner A. D. (2009). Posterior parietal cortex and episodic encoding: insights from fMRI subsequent memory effects and dual attention theory. *Neurobiol. Learn. Mem.* 91 139–154. 10.1016/j.nlm.2008.10.011 19028591 PMC2814803

[B52] Vilberg K. L. Rugg M. D. (2008). Memory retrieval and the parietal cortex: a review of evidence from a dual-process perspective. *Neuropsychologia* 46 1787–1799. 10.1016/j.neuropsychologia.2008.01.004 18343462 PMC2488316

[B53] Vincent J. L. Kahn I. Snyder A. Z. Raichle M. E. Buckner R. L. (2008). Evidence for a frontoparietal control system revealed by intrinsic functional connectivity. *J. Neurophysiol.* 2008;100 3328–3342. 10.1152/jn.90355.2008. *Erratum J. Neurophysiol.* 105:1427. 18799601 PMC2604839

[B54] Vincent J. L. Snyder A. Z. Fox M. D. Shannon B. J. Andrews J. R. Raichle M. E. (2006). Coherent spontaneous activity identifies a hippocampal-parietal memory network. *J. Neurophysiol.* 96 3517–3531. 10.1152/jn.00048.2006 16899645

[B55] Vulić K. Bjekić J. Paunović D. Jovanović M. Milanović S. Filipović S. R. (2021). Theta-modulated oscillatory transcranial direct current stimulation over posterior parietal cortex improves associative memory. *Sci. Rep.* 11:3013. 10.1038/s41598-021-82577-7 33542344 PMC7862221

[B56] Wagner A. D. Shannon B. J. Kahn I. Buckner R. L. (2005). Parietal lobe contributions to episodic memory retrieval. *Trends Cogn. Sci.* 9 445–453. 10.1016/j.tics.2005.07.001 16054861

[B57] Wang J. X. Rogers L. M. Gross E. Z. Ryals A. J. Dokucu M. E. Brandstatt K. L. (2014). Memory enhancement: targeted enhancement of cortical-hippocampal brain networks and associative memory. *Science* 345 1054–1057. 10.1126/science.1252900 25170153 PMC4307924

[B58] Watrous A. J. Tandon N. Conner C. R. Pieters T. Ekstrom A. D. (2013). Frequency-specific network connectivity increases underlie accurate spatiotemporal memory retrieval. *Nat. Neurosci.* 16 349–356. 10.1038/nn.3315 23354333 PMC3581758

[B59] Winocur G. Moscovitch M. (2011). Memory transformation and systems consolidation. *J. Int. Neuropsychol. Soc.* 17 766–780. 10.1017/S1355617711000683 21729403

[B60] Yeh N. Rose N. S. (2019). How can transcranial magnetic stimulation be used to modulate episodic memory: a systematic review and meta-analysis. *Front. Psychol.* 10:993. 10.3389/fpsyg.2019.00993 31263433 PMC6584914

